# Determination of the Frequency of Celiac Disease in Patients Presenting With Chronic Diarrhea

**DOI:** 10.7759/cureus.63638

**Published:** 2024-07-02

**Authors:** Amtiaz Ahmad, Ali Hyder, Attique Abou Bakr, Shujaat Hussain, Raja Taha Yaseen Khan, Muhammad Ali Khalid, Nasir Hassan Luck

**Affiliations:** 1 Department of Gastroenterology, Allama Iqbal Medical College, Jinnah Hospital, Lahore, PAK; 2 Department of Gastroenterology, Chandka Medical college, Shaheed Mohtarma Benazir Bhutto Medical University, Larkana, PAK; 3 Department of Gastroenterology, Pakistan Institute of Medical Sciences, Islamabad, PAK; 4 Department of Hepatogastroenterology, Sindh Institute of Urology and Transplantation, Karachi, PAK; 5 Department of Gastroenterology, King Abdulaziz Hospital, Jeddah, SAU

**Keywords:** lahore, jinnah hospital, gastroenterology, prevalence, chronic diarrhea, celiac disease

## Abstract

Introduction: Celiac disease (CD) is a chronic inflammatory disorder affecting mainly the digestive system and accounts for more than 50% of adult cases presenting to the gastrointestinal clinic with chronic diarrhea. Therefore, in our study, we aimed to determine the prevalence of CD in patients presenting with chronic diarrhea at the gastroenterology outpatient department of Jinnah Hospital, Lahore.

Methods: This cross-sectional study was conducted from December 9, 2021, to June 8, 2022, and included 140 patients aged 18 to 50 years with chronic diarrhea. Exclusion criteria were lack of informed consent and history of abdominal trauma or surgery. Data collected included age, gender, family history of CD, and clinical symptoms. Diagnostic measures involved serum tissue transglutaminase antibody IgA and IgG levels, endoscopy, and duodenal biopsy. Statistical analysis was performed using SPSS version 23 (IBM Corp, Armonk, NY), with a p-value of ≤0.05 considered significant.

Results: Among the 140 patients, 80 (57.14%) were males, with a mean age of 21 ± 4.35 years. Common symptoms included weight loss (73.5%), abdominal pain (20.7%), and stunted growth (5.7%). A family history of CD was reported in 14.29% of patients. Endoscopy findings included fissuring of the duodenal mucosa (77.9%), decreased height of duodenal folds (15.7%), and nodularity (6.4%). Histopathological examination revealed Marsh III b (65%), Marsh III c (21.4%), and Marsh III a (9.3%). CD was diagnosed in 23.57% of patients. Significant associations were found between CD and female gender, family history of CD, weight loss, stunted growth, and Marsh III c histopathology.

Conclusion: CD was diagnosed in 23.57% of patients with chronic diarrhea. It was more prevalent in females and those with a family history of CD. These findings emphasize the need for considering CD in the differential diagnosis of chronic diarrhea to ensure early detection and management.

## Introduction

Celiac disease (CD) is a significant cause of malabsorption, characterized as a chronic inflammatory enteropathy with flattened small bowel villi in genetically predisposed individuals [1]. The condition is triggered by the consumption of gluten-containing foods (such as wheat, rye, barley, and certain types of oats) and is exacerbated by environmental factors like interferon alpha (IFN-α) and infections. CD is now recognized as a multi-systemic disorder primarily affecting the digestive system [2,3]. A strict gluten-free diet (GFD) has been shown to improve both clinical symptoms and histological features in most patients with CD [4,5].

CD accounts for 56% of adult cases and 25% of pediatric cases of chronic diarrhea [6]. Diagnosing CD is challenging due to its presentation across various ages and a wide spectrum of symptoms [7,8]. A study by Ahmed AK et al. revealed that 25.9% of individuals with chronic diarrhea were indeed suffering from CD [9]. Similar studies by Parveen et al. and Bhavika et al. found that 35.5% and 16.17% of patients, respectively, had CD [10,11].

This study aimed to determine the prevalence of CD in patients presenting with chronic diarrhea in our community. It is important because many patients with chronic diarrhea receive only symptomatic treatment, and undiagnosed CD can lead to severe complications like intestinal lymphoma and increased mortality. The study’s findings will provide insights into the prevalence of CD among patients with persistent diarrhea in our community and highlight the need for increased awareness of CD as a potential underlying cause of chronic diarrhea.

## Materials and methods

Study design and study place

The Outpatient Department of Gastroenterology at Jinnah Hospital Lahore was the site of this cross-sectional study, which was conducted from December 9, 2021, to June 8, 2022. With a sample size of 140 patients, the study had a 95% confidence level, a 6% margin of error, and an estimated 16% prevalence of CD [10]. The sampling technique used was non-probability consecutive sampling.

Inclusion and exclusion criteria

The study comprised patients between the ages of 18 and 50 years, of any gender, and who presented with chronic diarrhea. Patients who did not provide informed consent or who had a history of abdominal trauma or surgery were excluded.

Data collection procedure 

Data regarding baseline variables such as age, gender, and family history of CD were collected for each patient. Venous blood samples were drawn for each patient and were sent to the hospital for complete blood count and determination of serum tissue transglutaminase antibody (tTG) IgA and IgG levels to confirm the diagnosis of CD. Endoscopy and duodenal biopsies were carried out in each patient and the biopsy specimen was then sent for histopathology.

Data analysis procedure

Utilizing SPSS version 23 software (IBM Corp, Armonk, NY), data analysis was performed. Mean and standard deviation were used for the expression of continuous variables while categorical variables were described as frequencies and percentages. A comparative analysis for continuous variables was performed using the student's t-test while that for categorical variables was done using the chi-square test. A p-value of ≤0.05 was considered to be significant.

## Results

A total of 140 patients with chronic diarrhea were enrolled in the study. Among them, 80 (57.14%) were males. The mean age was 21 ± 4.35 years. The most common presenting complaint associated with diarrhea was weight loss followed by abdominal pain and stunted growth noted in 103 (73.5%), 29 (20.7%), and 8 (5.7%), respectively. Family history of CD was noted in 20 (14.29%) (Table 1).

**Table 1:** Baseline characteristics of the studied population (n = 140) This table presents the mean ± standard deviation for continuous variables. The reference ranges for the continuous variables are as follows: hemoglobin: 13.5-17.5 g/dL for males, 12.0-15.5 g/dL for females; total leukocyte count (TLC): 4.0-11.0 (×10^9^/L); mean corpuscular volume (MCV): 80-100 fL; platelet count: 150-450 (×10^9^/L).

Study population		n (%)
Gender	Male	80 (57.14)
	Female	60 (42.86)
Symptoms	Weight loss	103 (73.5)
	Abdominal pain	29 (20.7)
	Stunted growth	8 (5.7)
Family history of celiac disease	Yes	20 (14.29)
	No	120 (85.71)
Endoscopic findings	Duodenal fissuring	109 (77.9)
	Nodularity of duodenal mucosa	9 (6.4)
	Decreased height of duodenal folds	22 (15.7)
Histopathological findings	Marsh III a	13 (9.3)
	Marsh III b	91 (65)
	Marsh III c	30 (21.4)
	Normal	6 (4.3)
Celiac disease	Yes	33 (23.57)
	No	107 (76.43)
Mean age (years ± SD)		21 ± 4.35
Hemoglobin (g/dL)		10.1 ± 2.6
MCV (fL)		72.8 ± 4.3
Total leucocyte count (x10^9^/L)		8.9 ± 5
Platelet count (x10^9^/L)		264 ± 51

On endoscopy, fissuring of duodenal mucosa was noted in 109 (77.9%) patients while 22 (15.7%) patients had decreased height of duodenal folds, and nodularity of duodenal mucosa was observed in 9 (6.4%) patients. On histopathology, the most common finding was Marsh III b followed by Marsh III c and III a noted in 91 (65%), 30 (21.4%), and 13 (9.3%) patients, respectively. Out of 140 patients, CD was diagnosed in 33 (23.57%) patients (Table 1).

Mean hemoglobin was 10.1 ± 2.6 g/dL, mean corpuscular volume was 72.8 ± 4.3 fL, total leukocyte count was 8.9±5 x 10^9^/L, and platelet count was 264 ± 51 x 10^9^/L. On comparative analysis, female gender, family history of CD, symptoms of weight loss, stunted growth, and Marsh III c on histology were the factors significantly associated with the presence of CD in patients with chronic diarrhea (Table 2).

**Table 2:** Stratification of baseline variables to determine their association with celiac disease χ^2^*, *chi-square.

Variables		Celiac disease		χ^2 ^value	p-Value
		Yes (n = 33), N (%)	No (n = 107), N (%)		
Gender	Male	14 (42.4)	66 (61.7)	3.74	0.05
	Female	19 (57.6)	41 (38.3)		
Symptoms	Weight loss	24 (72.7)	79 (73.8)	5.24	0.034
	Abdominal pain	3 (9.1)	26 (24.3)		
	Stunted growth	6 (81.8)	2 (18.7)		
Family history of celiac disease	Yes	18 (54.5)	2 (1.9)	8.81	≤0.001
	No	15 (45.5)	105 (98.1)		
Endoscopic findings	Duodenal fissuring	24 (72.7)	85 (79.5)	2.21	0.078
	Nodularity of duodenal mucosa	6 (18.1)	3 (2.9)		
	Decreased height of duodenal folds	3 (9.2)	19 (17.6)		
Histopathology	Marsh III a	3 (9.1)	10 (9.4)	11.34	≤0.001
	Marsh III b	26 (78.8)	65 (60.7)		
	Marsh III c	4 (12.1)	26 (24.3)		
	Normal	0 (0)	6 (5.6)		

## Discussion

CD affects approximately 0.5% to 1% of the global population and is often underdiagnosed [1,12,13]. The increasing adoption of Western diets, which frequently include gluten-containing foods such as bread and noodles, could lead to a rise in CD incidence in Asia [14]. The majority of CD patients have the HLA-DQ2 and HLA-DQ8 genotypes, which, in conjunction with immune system cells, lead to immune-mediated enteropathy and small intestinal malabsorption. CD symptoms are varied, encompassing gastrointestinal issues like diarrhea, bloating, and steatorrhea, as well as extra-intestinal symptoms including anemia, weight loss, dermatitis herpetiformis, and osteoporosis [14]. The interaction between genetic, immunological, and environmental factors results in a broad spectrum of CD presentations. Additionally, CD is more prevalent among individuals with certain genetic syndromes such as Williams syndrome, Down syndrome, and Turner syndrome, as well as autoimmune conditions like thyroiditis and liver disease [15].

The prevalence of CD in the Asia-Pacific region is reported to be between 0.32% and 1.41% in children and 0.22% and 1.22% in adults, according to Ashtari and his colleagues [16]. Diagnosing CD can be challenging due to its diverse clinical manifestations. While serologic testing is crucial, it can sometimes be overlooked due to the variability in symptoms [17]. Despite increased diagnosis rates over the past 30 years, many cases remain undiagnosed [18]. Diagnostic methods recommended by the British Society of Gastroenterology (BSG) and the American College of Gastroenterology (ACG) include intestinal biopsies, total IgA levels, and serum IgA anti-tissue transglutaminase antibody (anti-tTG) tests for patients over the age of two who are suspected of having CD [19,20]. The World Gastroenterological Association suggests that in low-resource settings, a simple anti-tTG IgA test along with endoscopic morphological observations may suffice for diagnosis [21].

A study by Rampertab et al. analyzed CD patient data from 1952 to 2004, noting that the mean age at diagnosis was 17.4 and 43.3 years, and 25% had a family history of CD [22]. The time from symptom onset to diagnosis varied from 4.6 to 7.5 years, with diarrhea being the most common symptom in 46.7% of patients. The study observed a decline in the proportion of patients presenting with diarrhea over time, from 91.3% before 1981 to 37.2% after 2000 [9]. Green's comprehensive study further highlighted the diverse range of clinical symptoms experienced by CD patients [23].

Research from the Columbia University Celiac Center examined 227 biopsy-confirmed CD patients, finding a higher prevalence in women and a decrease in mean symptom duration from 9.0 years to 4.4 years before and after 1993, respectively [19]. Similar to these findings, our study observed a higher prevalence of CD among females and patients with a positive family history of the disease.

The limitations of this study include the relatively small sample size and the focus on a single hospital, which may not be representative of the broader population. Additionally, the study's cross-sectional design limits the ability to establish causality. Future research with larger, multicenter cohorts is necessary to confirm these findings and to provide a more comprehensive understanding of the prevalence and demographic patterns of CD in patients presenting with chronic diarrhea.

## Conclusions

This study demonstrates a notable association between chronic diarrhea and CD, indicating that CD should be considered in patients with chronic diarrhea. It also found a higher prevalence of CD among female patients and those with a family history of the condition. These findings emphasize the importance of considering demographic and familial factors in the diagnostic process. Comprehensive diagnostic protocols, including serologic testing and, when necessary, endoscopic and histopathological examinations, are essential for accurate and timely diagnosis. Early detection and adherence to a strict GFD can prevent severe complications associated with untreated CD. Despite its limitations, such as small sample size and single-hospital focus, this study highlights the need for increased clinical awareness and thorough investigation of CD in patients presenting with chronic diarrhea to improve patient outcomes through early diagnosis and appropriate dietary management.
